# Improving Compliance With GASS (Glasgow Antipsychotic Side Effects Scale) Monitoring for Patients Receiving Depot Antipsychotics at Swale CMHT : A Two-Cycle Clinical Audit

**DOI:** 10.1192/bjo.2026.11687

**Published:** 2026-06-30

**Authors:** Neelima Liz John

**Affiliations:** Kent and Medway Mental Health NHS Trust, Kent, United Kingdom

## Abstract

**Aims:**

Systematic monitoring of antipsychotic side effects is essential for medication adherence and relapse prevention. To audit compliance with recommended GASS monitoring for patients receiving depot antipsychotic injections in a community mental health team (CMHT). Local Trust and NICE guidelines recommend use of validated rating scales such as the Glasgow Antipsychotic Side-Effect Scale (GASS), with completion at one month after initiating depot antipsychotics and at least every six months thereafter

**Methods:**

Using a retrospective review, 20 randomly selected patients receiving depot antipsychotics at Swale CMHT were assessed. Electronic notes (Rio) and uploaded specialist assessment forms were examined to determine whether GASS assessments were completed at the minimum six-monthly interval. Initial one-month post-initiation GASS assessments were excluded as most service users had commenced treatment prior to the first audit cycle.

**Results:**

The 1st cycle of the audit was conducted from Nov 1^st^ 2023 to Jan 30^th^2024 and six-month GASS monitoring were reviewed between Jan 2022 till Oct 2023. The 2nd cycle of the audit was done between 15^th^Jan 2025 to March 30^th^and six-month GASS monitoring were reviewed between Jan 2024 till Dec 2024.

In the first audit cycle, only 5% (1/20) of service users had GASS assessments completed at the recommended six-monthly interval. Following staffing improvements, including new pharmacy team joining and increased awareness of guidelines, the re-audit demonstrated substantial improvement: 75% (15/20) of service users received GASS assessments every six months. Reasons for missed assessments were often undocumented.

**Conclusion:**

The initial audit cycle identified a significant deficit in the objective monitoring of antipsychotic side effects using the Glasgow Antipsychotic Side-effect Scale (GASS). Following the implementation of increased awareness, and optimized staffing levels, the second cycle demonstrated a marked improvement in compliance. However, documentation gaps and patient refusals persist. These findings highlight the need for further targeted interventions to ensure full adherence to Trust clinical guidelines.

